# Enhancing Rubber Vulcanization Cure Kinetics: Lowering Vulcanization Temperature by Addition of MgO as Co-Cure Activator in ZnO-Based Cure Activator Systems

**DOI:** 10.3390/polym16070876

**Published:** 2024-03-22

**Authors:** Md Najib Alam, Vineet Kumar, Seok U Jeong, Sang-Shin Park

**Affiliations:** 1 School of Mechanical Engineering, Yeungnam University, 280, Daehak-ro, Gyeongsan 38541, Republic of Korea; mdnajib.alam3@gmail.com (M.N.A.); vineetfri@gmail.com (V.K.); 2 Graduate School of Mechanical Engineering, Yeungnam University, 280, Daehak-ro, Gyeongsan 38541, Republic of Korea; fffu123@naver.com

**Keywords:** rubber, cure activator, magnesium oxide, zinc oxide, vulcanization kinetics

## Abstract

Vulcanization is a chemical modification of rubber that requires a considerable amount of thermal energy. To save thermal energy, the kinetics of rubber vulcanization should be improved. In this article, the curing properties of rubber vulcanization are thoroughly investigated using the moving die rheometer (MDR) technique. To enhance the kinetics in different stages of ZnO-based sulfur vulcanization systems, small amounts of MgO were added. The results revealed that the small amount of 1 to 2 phr (per hundred grams of rubber) of MgO in the controlled 5 phr ZnO-based curing systems can significantly improve the curing kinetics. For example, the optimum curing time of 1 phr MgO added to the 5 phr ZnO-containing semi-efficient vulcanization system at different temperatures was more than half that of the controlled 5 phr ZnO-only compound. While maintaining a similar rate of vulcanization, the vulcanization temperature can be reduced by up to 20 °C by using MgO as a co-cure activator, which exhibits similar or better rheometric mechanical properties compared to the controlled compounds. With the addition of MgO as a co-cure activator, the vulcanization reactions become very fast, enabling vulcanization to be completed, even at the boiling point of water (100 °C) with an affordable curing time (<1 h). By reducing the vulcanization temperature, the scorch safety time can be enhanced in the ZnO/MgO-based binary cure activator-containing vulcanizates. Overall, MgO could be a potential candidate as a co-cure activator with ZnO for the vulcanization of rubber, offering better economical and eco-friendly methods.

## 1. Introduction

Among different polymeric materials, rubber has wide industrial applications [1]. However, raw rubber has limited applications because it is soft in hot weather and stiff in cold weather. To maintain uniformity in mechanical properties with variable environmental temperatures, raw rubber must be processed. The processing of rubber is performed via curing, which produces cross-linking between the rubber chains, transforming individual rubber macromolecules into a connected network that can sustain its mechanical properties over a wider temperature range and deformation [2]. Many rubber products, as we see in our daily lives, are processed rubber.

After the invention of the cross-linking process known as vulcanization of rubber by Charles Goodyear in 1839 through simple heating of rubber and sulfur, numerous curing ingredients have been developed to reduce the vulcanization time and enhance cure efficiency [3,4]. Currently, sulfur-based rubber vulcanization is the most common method, generally performed at 140–150 °C, with the required curing time depending on the type of rubber and the efficiency of the accelerator, which can vary from 10 to 30 min.

Recently, global warming has become a challenging issue, and the minimum use of thermal energy is recommended, along with reduced reliance on fossil fuels [5]. In the rubber industry, the use of thermal energy can be minimized by enhancing the curing rate without increasing vulcanization temperature and time. This is only achievable by selecting faster curing ingredients for rubber vulcanization. While higher temperatures can enhance the curing rate, they may also degrade important properties [6]. Additionally, at elevated temperatures, the rate of release of environmentally polluting gases such as H_2_S, CS_2_, and secondary amines from vulcanized rubber, as byproducts can increase [6,7]. Therefore, reducing vulcanization temperature is urgently needed from both economic and environmental perspectives.

In rubber vulcanization, accelerators and activators play a key role in the kinetics of the process. A very fast curing system can reduce the vulcanization time, but it may have drawbacks such as low scorch safety and an increased reversion rate [8]. These properties significantly affect the vulcanizate properties, leading to improper vulcanization throughout the rubber matrix. It has been found that lowering the vulcanization temperature can enhance scorch safety and other mechanical properties, although it takes a longer time to cure and may not be economically favorable. Currently, thiuram-type accelerators combined with ZnO-based cure activators serve as a conventionally faster curing system for rubber vulcanization. Recently, Alam et al. [9,10] observed that cure activities, especially the cure rate, can be further enhanced by using MgO as a single or co-cure activator with ZnO in rubber vulcanization. Although the optimum curing time, rate of cross-linking, and certain mechanical properties were enhanced, they had very low scorch safety and reduced cross-link density, possibly due to fast initiation of the cross-linking reaction and increased reversion [9,10]. Hence, new opportunities can arise by enhancing curing kinetics to reduce vulcanization temperature, achieving better scorch safety, reduced reversion rate, and improved cross-linking. Investigation shows that ZnO has many important roles in different stages of accelerated sulfur vulcanization [11]. Based on the previous reports, it was evident that either ZnO or zinc-based compounds are necessarily important to obtain vulcanizates with industrial value [12,13]. After ZnO, MgO has recently gained much attention from rubber scientists because of its inexpensive, abandoned, low toxicity, and high potential to enhance the kinetics of vulcanization in different rubber systems [14,15,16,17,18,19,20,21]. Most importantly, it was found that ZnO and MgO might undergo synergistic activities during accelerated sulfur vulcanization [10] and can enhance the kinetics as well as the mechanical properties.

Kinetic studies are necessarily important to understand the overall vulcanization reaction. A few important techniques, such as differential scanning calorimetry (DSC) and oscillating disc rheometer (ODR) tests, are employed to study the kinetics of vulcanization [22]. The main drawbacks of DSC studies include their limited ability to accurately predict the induction period, particularly for lower exothermic vulcanization kinetics [23]. However, ODR is considered best because, along with kinetic data, it also provides certain mechanical properties of the vulcanized rubber. The ODR curve is based on considering stiffness, which is directly proportional to the degree of vulcanization compound versus time of vulcanization. Based on the ODR curve, Coran proposed a model for accelerated sulfur vulcanization where four main kinetic steps can be involved [24,25]. In the first kinetic step, a sulfurating complex is formed. In the second step, cross-linking precursors are formed, followed by activation of the sulfurating complex. In the third step, polysulfide rubber networks are formed. In the fourth and final step, these polysulfides turn into mature cross-links [22,24,25,26]. The general schematics of rubber vulcanization, considering the kinetic model of accelerated sulfur vulcanization, can be found elsewhere [11,22].

Studies by Alam et al. [10], Zhao et al. [27], and Hadi et al. [28] have revealed the potential role of cure activators such as zinc oxide in the kinetics of rubber vulcanization. They investigated the influence of zinc oxide on the cure activity of natural rubber-based curing systems. Their findings suggest that the curing rate can decrease after reaching an optimum level (1–2 g per hundred grams of rubber) of zinc oxide in efficient vulcanization (EV) and semi-efficient vulcanization (SEV) systems [10,27]. However, for conventional vulcanization systems, the curing rate may increase with higher amounts of zinc oxide (>5 phr) [28]. While the rate of vulcanization may decrease with certain vulcanization systems, higher amounts of zinc oxide can lead to the production of more cross-link precursors, thereby enhancing cross-link density and mechanical properties [27].

Previous studies by Alam et al. [9,10] concluded that the level of ZnO can be substantially reduced by utilizing zinc-based binary accelerator systems or employing MgO/ZnO binary cure activator systems. These approaches lead to improved vulcanization kinetics at static temperature, as well as enhancements in tensile strength, fracture toughness, and elongation at break properties. However, such reductions in ZnO levels come at the cost of compromised cross-link density and mechanical modulus values [9,10]. Currently, although the level of zinc oxide can be decreased in many rubber formulations [29,30,31,32,33,34,35,36,37,38], for certain applications like heavy-duty truck tire manufacturing, the amount cannot be compromised. These applications require a high degree of cross-linking to achieve elevated modulus values and low heat build-up properties [11]. From this perspective, reducing the vulcanization temperature while enhancing the vulcanization kinetics could prove to be an effective approach for reducing environmental pollution, production costs, and overall performances. While previous studies [9,10] explored the possibility of reducing ZnO levels using MgO as a co-cure activator, detailed kinetic studies necessary for potential reductions in vulcanization temperature remain unexplored. Lowering the vulcanization temperature with improved curing kinetics not only decreases production costs but also enhances the quality of rubber products, safeguarding them from issues like high-temperature scorching and reversion due to overcuring, particularly in thicker rubber products.

In this paper, our objective is to reduce the vulcanization temperature with an affordable time in accelerated sulfur vulcanization without the loss of vulcanizate properties, especially the level of rheometric mechanical properties. To improve the kinetics of rubber vulcanization, MgO was utilized as a secondary cure activator in the ZnO-based cure activator systems. To understand the mechanism of sulfur cross-linking in the presence of MgO and ZnO co-cure activators, different vulcanization systems, namely EV and SEV systems with high to moderate accelerator-to-sulfur ratios, are chosen. Because cure activators primarily form accelerator–activator complexes, a higher concentration of accelerator can facilitate a better understanding of the mechanism of accelerated sulfur vulcanization. It is believed that MgO plays a critical role in the in situ formation of zinc accelerator complexes, and their activity and amounts depend on accelerator-to-sulfur ratios. The curing studies were conducted followed by the ODR technique with different vulcanization temperatures ranging from 100 to 140 °C. The different kinetic parameters have been correlated with Arrhenius-type relationships to investigate the role of MgO in the activation energies.

## 2. Materials and Methods

### 2.1. Materials

Natural rubber (NR, RSS-3) and the curing additives such as zinc oxide, stearic acid, accelerator tetramethyl thiuram disulfide (TMTD), and sulfur were obtained from the Thai Rubber Research Institute, Bangkok, Thailand. Magnesium oxide light in fine powder form was purchased from AppliChem PanReac, Bangkok, Thailand.

### 2.2. Mixing of Curing Ingredients with Rubber

The mixing of curing ingredients was performed at room temperature in a laboratory-size open two-roll mill. The speeds of the front and rear rolls were fixed at 20 and 24 rpm, respectively, to maintain a 1.2 friction ratio. Initially, raw rubber was masticated for five minutes with a 2 mm nip gap between the two rolls. Then, cure activators (ZnO, MgO, and stearic acid) were added and milled for another five minutes, reducing the nip gap to 1 mm. After that, accelerator (TMTD) and sulfur were added together and milled for another five minutes. The mixing time was fixed at 15 min for all compounds. The compositions of rubber compounds are provided in Table 1. Different curing systems can be identified from the abbreviation in the mix composition and can be realized from their respective accelerator-to-sulfur ratios: 4.32 for EV and 0.96 for SEV systems, as presented in Table 1. For example, the EV/5-ZnO/1-MgO compound belongs to the EV system, where 5 phr ZnO along with 1 phr MgO were used as cure activators.

### 2.3. Measurement of Curing Properties

Curing properties of the compounded rubber were investigated by a Moving Die Rheometer (MDR, Rheo Tech MD+, Model no. A022S, MonTech USA, Columbia City, IN, USA). The instrument provides a torque versus time plot at a constant vulcanization temperature, from which rheometric mechanical properties, such as minimum torque (M_L_), highest torque (M_H_), ΔM (M_H_ − M_L_), torque after 300 s of overcuring (M_300_), and percentage of reversion in torque at 300 s of overcuring (R_300_), as well as kinetic parameters such as scorch time (t_2_, considering 2% progress on Δ torque), optimum curing time (t_90_, considering 90% progress on Δ torque), the actual rate of cross-linking {R_v_ = (Mt_90_ − Mt_2_)/(t_90_ − t_2_)}, and the cure rate index {CRI = 100/(t_90_ − t_2_)}, were obtained. The different curing data are presented in the Supplementary Materials (Tables S1–S5).

The kinetic parameters, such as scorch safety time and the rate of cross-linking values, were fitted with the Arrhenius equation of temperature-dependent rate as follows:

$$k=Ae^{-\frac{E_{a}}{RT}}$$

where k denotes the rate of a process, A is the pre-exponential factor, E_a_ is the energy of activation for that process, R is the universal gas constant, and T is the temperature in absolute scale. Taking the logarithm, the above equation turns into:

$$\ln k=\ln A-\frac{E_{a}}{RT}$$

Replacing k as t_2_ and R_v_ at different temperatures, different E_a_ values can be obtained. Since t_2_ is represented as time, its inverse can be regarded as rate and follows the above equation very precisely. On the other hand, R_v_ is the true rate of vulcanization; hence, the E_a_ value obtained can be regarded as the activation energy of vulcanization.

## 3. Results and Discussion

**Figure 1:**
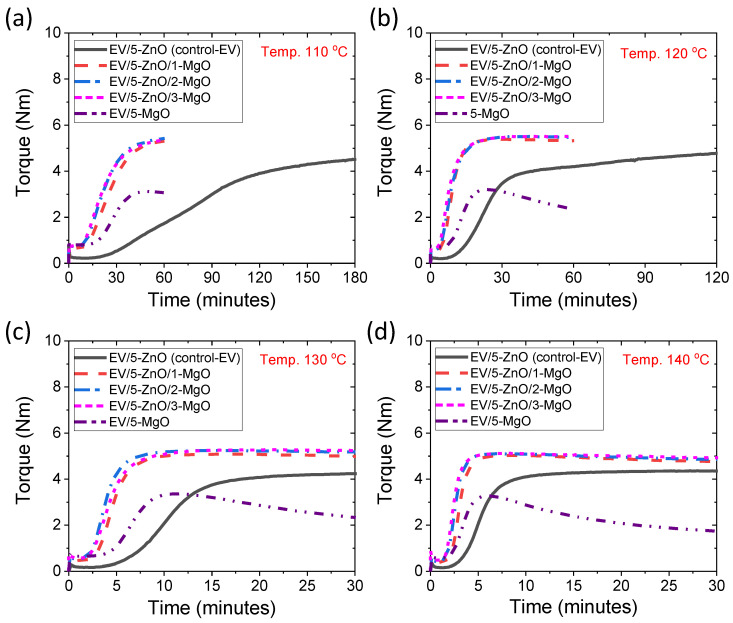
Rheographs for EV curing systems at different vulcanization temperatures; (**a**) 110 °C, (**b**) 120 °C, (**c**) 130 °C, and (**d**) 140 °C.

Cure curves (rheographs) of EV systems with different types of cure activator at different temperatures are shown in Figure 1a–d. In the rheographs, three distinct zones, such as induction, cure, and overcure, can be clearly identified [22]. It can be seen that the induction and cure zones of ZnO-only compounds can be shortened by the addition of a small amount of MgO as a secondary cure activator. This suggests that MgO has a considerable effect on the kinetics of rubber vulcanization. With increasing vulcanization temperatures from 110 °C to 140 °C, the different time zones are further shortened, and the overcure zones show lower reversion resistances. Tables S1–S5 provide the reversion properties concerning M_300_ and R_300_. It is evident from the tables that the M_300_ value decreases gradually with increasing vulcanization temperature, while the R_300_ values remain notably low for hybrid cure activator systems at lower temperatures. Therefore, implementing low-temperature vulcanization is desirable to achieve improved reversion resistance in rubber when utilizing binary cure activator systems. It can also be found from the rheographs that the ZnO-only compound shows a slight marching tendency in the overcure zone, whereas the MgO-only compound has a higher reversion tendency in the overcure zone. However, in the presence of both cure activators, ZnO and MgO, a perfect equilibrium cure curve can be obtained in the overcure zones. A better equilibrium curve might indicate better age-resistant rubber since it has no change in the degree of cross-linking with time and temperature after complete curing [39,40].

**Figure 2:**
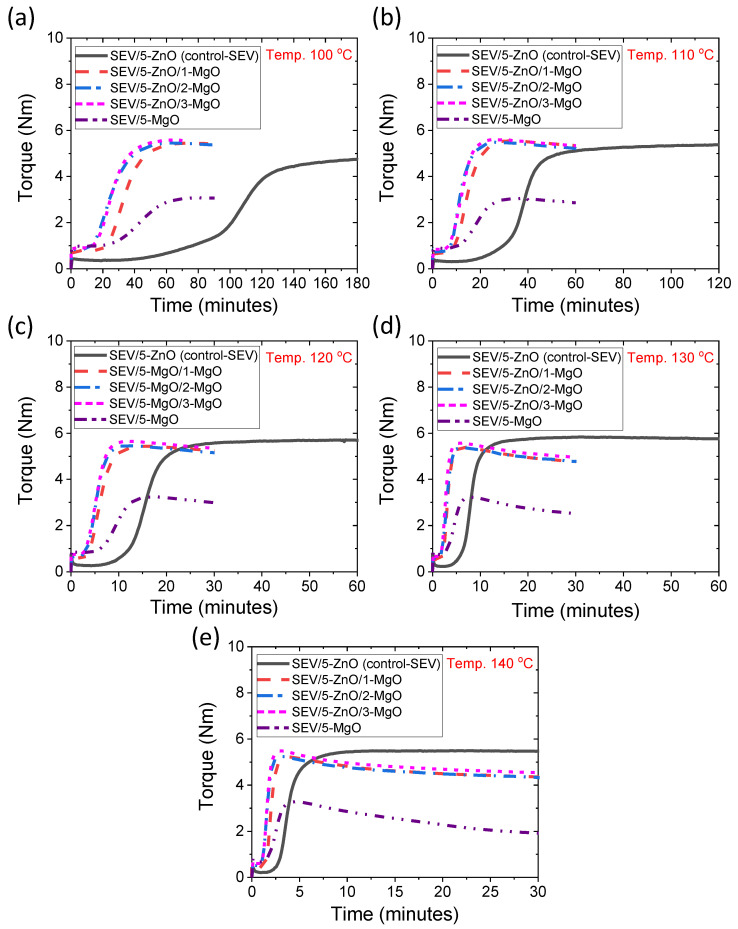
Rheographs for SEV curing systems at different vulcanization temperatures; (**a**) 100 °C, (**b**) 110 °C, (**c**) 120 °C, (**d**) 130 °C, and (**e**) 140 °C.

Rheographs for different SEV curing systems are provided in Figure 2a–e. Rheographs of SEV curing systems are similar to those for EV curing systems. However, higher values in the rheometric torque after curing have been found for SEV vulcanization systems compared to EV curing systems. Also, the tendency of reversion in the overcure zone is more pronounced in the SEV curing systems compared to EV curing systems, and the reversion rates are increased with increasing vulcanization temperature (Tables S1–S5). The elevated reversion rates observed in SEV curing systems might result from the formation of increased quantities of polysulfide linkages in the presence of a lower concentration of accelerator–activator complexes, as opposed to the higher concentration observed in EV curing systems. Polysulfide linkages have a tendency to form cyclic sulfides, which can break the linear sulfur bridges during prolonged heating, thereby impacting the reduction in rheometric torque values [8,39].

**Figure 3:**
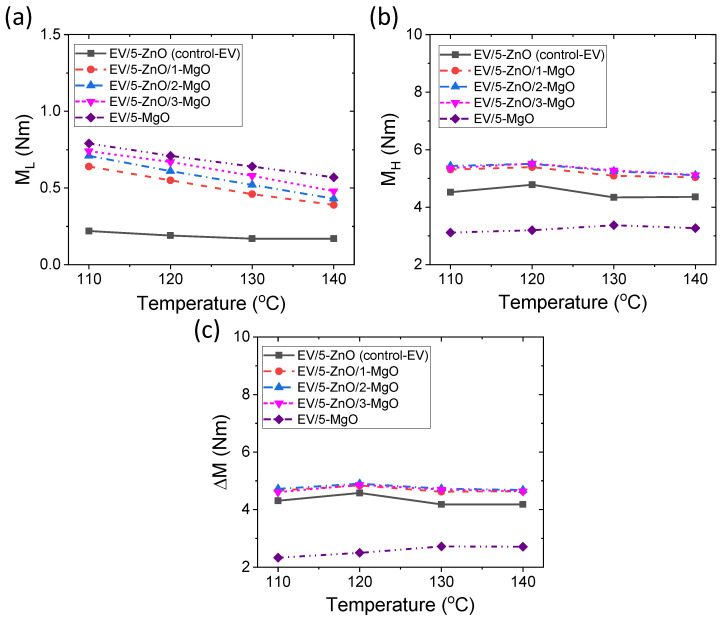
Rheometric mechanical properties for EV curing systems; (**a**) M_L_, (**b**) M_H_, (**c**) ΔM.

Rheometric mechanical properties are important for predicting final vulcanizate properties. For example, torque is proportional to stiffness, and ΔM is proportional to cross-link density [22]. The different mechanical properties of the EV curing systems are provided in Figure 3a–c. From Figure 3c, the lowest torque (M_L_) gradually decreases with increasing temperature for all EV curing systems. It is interesting to note that by increasing the amounts of MgO in the systems, M_L_ values can be enhanced. This might indicate that a higher amount of accelerator can be grafted to the rubber chains, resulting in higher viscosity in the compound incorporating MgO, which decomposes the accelerator and attaches to the rubber chains more effectively than ZnO [9,10]. From Figure 3b, it can be seen that the MgO-only compound shows the lowest values in the M_H_, and binary cure activators containing compounds have higher M_H_ values. It is believed that when MgO interacts with the accelerator, it forms Mg-dithiocarbamate, which initially converts polysulfide cross-links into cyclic sulfides. This process reduces the number of cross-links, contrasting with the in situ formation of Zn-dithiocarbamate [9,10,39]. Consequently, MgO-only compounds exhibit the lowest M_H_ value compared to other activator systems. The ΔM values in Figure 3c follow a similar trend as M_H_ values. It can be seen from Figure 3b,c that M_H_ and ΔM can slightly increase with temperature and then decrease. This could be due to improved interactions between the curatives through sulfur diffusion [41] above the melting point of elemental sulfur near 110 °C; then the decrease might be due to reduced efficiency of the curing reagent at elevated temperatures. The improved ΔM in the binary activator systems could be advantageous in mechanical properties compared to ZnO as a single cure activator in the EV curing system.

**Figure 4:**
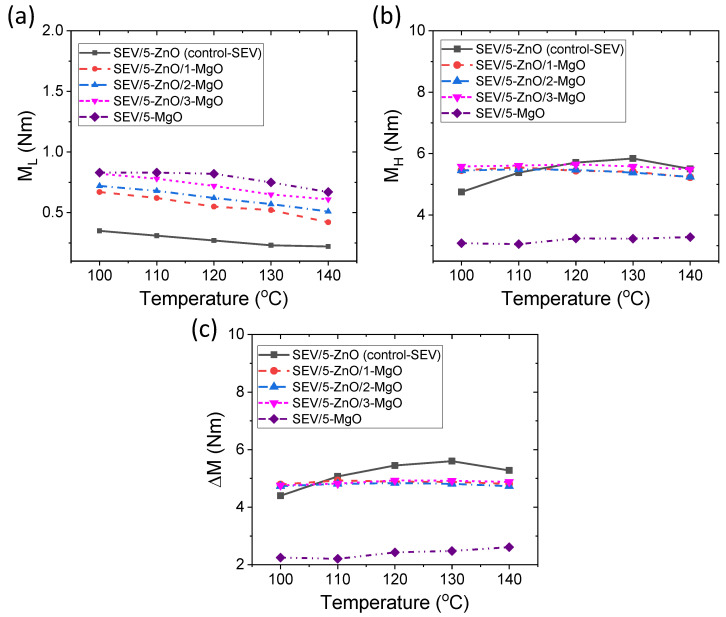
Rheometric mechanical properties for SEV curing systems; (**a**) M_L_, (**b**) M_H_, (**c**) ΔM.

The different rheometric mechanical properties for SEV curing systems are provided in Figure 4a–c. The M_L_ values for both SEV and EV curing systems follow a similar behavior with vulcanization temperature (Figure 4a). The M_L_ values are slightly higher in SEV curing systems than in EV curing systems, which could be due to the higher molecular weight of cross-link precursors attached to rubber chains with an increased number of sulfur atoms in the former cases than in the latter cases. From Figure 4b, it is evident that M_H_ values are better for binary cure activator compounds at lower temperatures compared to ZnO-only compounds. The ZnO-only compound shows the best value at 130 °C of vulcanization temperature. From Figure 4c, it is evident that ΔM values remain unchanged with increasing vulcanization temperature for MgO-containing binary cure activator compounds. However, for the ZnO-only compound, ΔM values increase up to 130 °C and then decrease with further increasing temperature. From this observation, it can be concluded that in the presence of MgO as a co-cure activator, the achieved cross-link density may become independent of temperature increase, and vulcanization can be achieved at low temperatures, even below the melting point of sulfur. The constant ΔM for MgO-added vulcanizates at low temperatures may suggest that a better and constant amount of cross-link precursors may form at fixed ZnO contents and undergo final cross-links with more specific sulfur-ranked cross-links such as disulfide cross-links [17]. On the other hand, the ZnO-only compound forms an increased amount of cross-link precursor with increasing temperature and provides increased ΔM up to 130 °C. Hence, from a mechanical point of view, vulcanization can be possible at very low temperatures without loss in mechanical properties and the degree of cross-linking using MgO as a co-cure activator along with ZnO.

**Figure 5:**
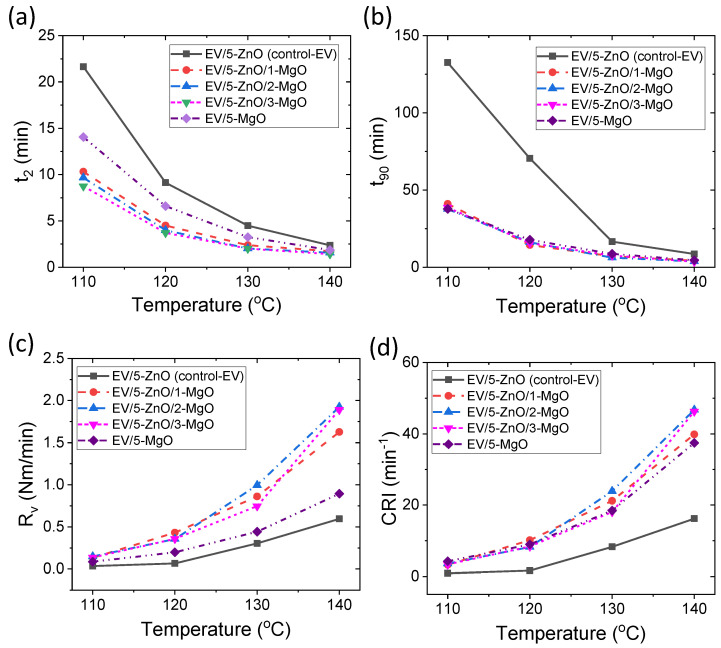
Curing properties for EV curing systems; (**a**) t_2_, (**b**) t_90_, (**c**) R_v_, and (**d**) CRI.

The different curing parameters for EV curing systems are plotted in Figure 5a–d. From Figure 5a, MgO-containing compounds have lower t_2_ compared to ZnO-only compounds. It is interesting to note that MgO and ZnO-based binary cure activator-containing compounds have much lower t_2_ values than both MgO and ZnO as single cure activator compounds. This result may indicate that MgO and ZnO-based in-situ compounds may interact to form a more reactive sulfurating complex [9,10]. From this figure, it can also be evident that t_2_ reduction occurs more rapidly in ZnO-only compounds than in MgO-containing compounds with increasing vulcanization temperature. Figure 5b represents the variation in the optimum curing times with different temperatures. From this figure, it can be found that the t_90_ value decreases more rapidly for the control-EV system than for all other MgO-containing compounds. It can also be seen that t_90_ is more than double compared to MgO-containing compounds. Hence, a catalytic amount of MgO could be effective in reducing the vulcanization time more economically. From Figure 5c, the rate of vulcanization is much lower for the control-EV system compared to MgO-added vulcanizates. With increasing vulcanization temperature, the rate increases more rapidly in ZnO- and MgO-based binary cure activator systems compared to single cure activator systems. It is evident that the addition of MgO to the binary cure activator-containing compounds can result in more than doubling of the vulcanization rate (R_v_) compared to the control-EV system. Figure 5d suggests that CRI values similarly increase with rising temperature. It is important to note that a higher CRI value only signifies the completion of the first stage of vulcanization, not the actual rate of vulcanization. Therefore, while the R_v_ values of ZnO-only and MgO-only compounds may not differ significantly, their CRI values vary considerably. This is because the MgO-only compound achieves a lower ΔM value within a shorter period of time, resulting in a higher CRI value.

**Figure 6:**
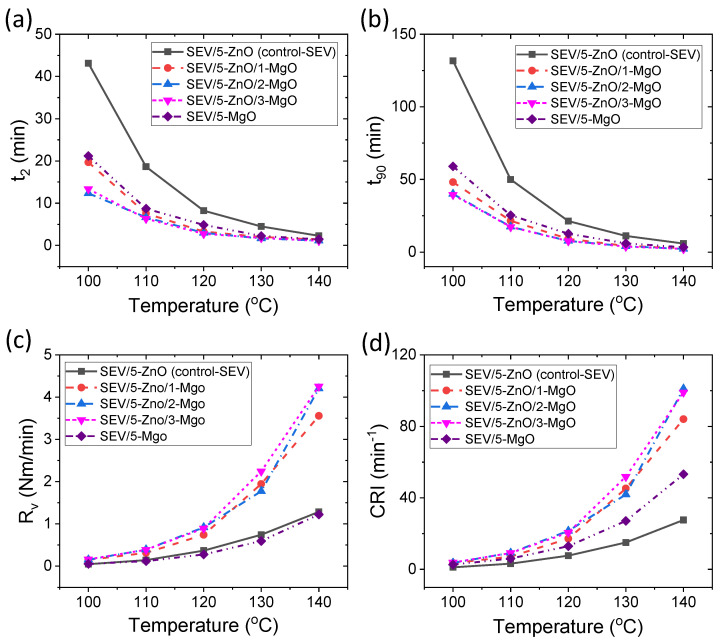
Curing properties for SEV curing systems; (**a**) t_2_, (**b**) t_90_, (**c**) R_v_, and (**d**) CRI.

Different curing parameters for SEV curing systems are provided in Figure 6a–d. From Figure 6a, t_2_ of different SEV compounds shows a similar behavior with temperature as EV curing systems. However, there is an advantage in better t_2_ values in SEV curing systems at lower temperatures compared to EV curing systems. From Figure 6b, it is evident that t_90_ values for different SEV curing systems also follow a similar behavior with increasing temperature as EV curing systems. It is interesting to note that MgO does not have a significant effect on t_90_ values after 1 phr in EV curing systems, but it can reduce the t_90_ value up to 2 phr in SEV curing systems. Hence, only 1 or 2 phr of MgO could be sufficient for extensive reduction in the curing time. From Figure 6c, R_v_ values of different SEV curing systems follow a similar behavior as EV curing systems with temperature but with higher R_v_ values in the former curing systems than in the latter curing systems. Figure 6d illustrates the CRI values in SEV curing systems. These values exhibit similar trends with temperature and are influenced by the nature of the cure activators. Interestingly, the rate of vulcanization in MgO-only compounds is lower compared to ZnO-only compounds at various temperatures in SEV curing systems, contrary to the behavior observed in EV curing systems. Therefore, it can be concluded that MgO-only acts as a less effective cure activator for achieving a faster vulcanization rate. However, it accelerates the intermolecular interactions, as evidenced by the CRI values, thereby reducing the vulcanization time. From Figure 5b,c and Figure 6b,c, binary cure activator-containing compounds have similar t_90_ and R_v_ values at 120 °C of vulcanization temperature compared to the control compounds at the vulcanization temperature of 140 °C. Hence, at least 20 °C of vulcanization temperature can be reduced by the addition of just 1 or 2 phr of MgO to the binary cure activator-containing vulcanizates.

**Figure 7:**
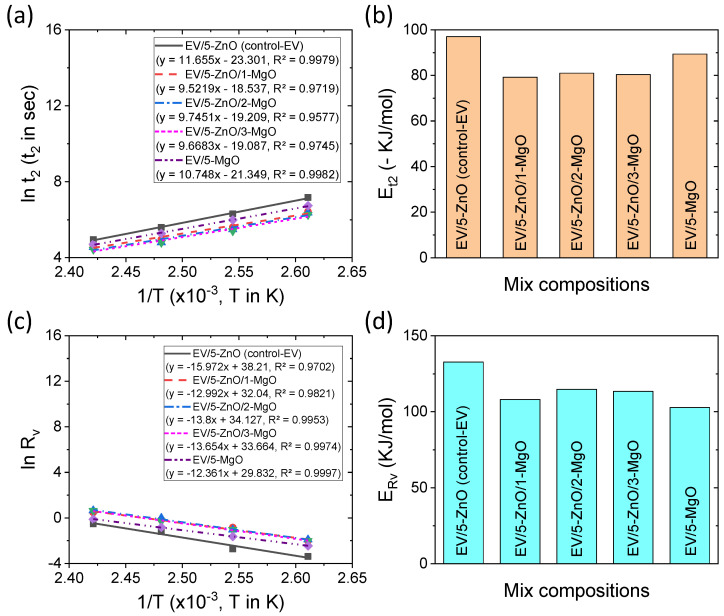
Arrhenius plots for activation energies of EV curing systems: (**a**) Arrhenius plot with respect to scorch safety time, (**b**) activation energy for cross-link precursor formation, (**c**) Arrhenius plot with respect to true cross-linking rate, and (**d**) activation energy of cross-linking formation.

According to the Coran kinetic model [22,23,24], there is a rate involved in the formation of cross-link precursors from the active sulfurating complex. Since the formation of cross-link precursors does not provide increasing torque, the rate cannot be calculated directly [22]. However, the time of formation of the cross-linking precursors should be lower than the scorch safety time. Here we consider scorch safety time as just only 2% achievement of the full torque (ΔM); hence, the scorch safety time, which is a specific point, can be roughly considered to correlate with the rate of formation of cross-link precursor. As the rate formation of cross-links by the activation of cross-link precursors may occur according to the Coran kinetic model [22,23,24], the rate of formation of cross-links can be used to determine the activation energy of the cross-linking reaction. Different plots on Arrhenius equations with respect to t_2_ and R_v_ and their corresponding activation energies for EV curing systems are provided in Figure 7a–d. From Figure 7a, it can be seen that the plots are very linear and hence can be useful to predict the scorch safety time at other temperatures beyond the experimental temperatures. From the activation energy plots in Figure 7b, it can be concluded that the addition of MgO can reduce the activation energy of the formation of cross-link precursors. This could be due to faster decomposition of accelerator by MgO that can enhance the rate of formation of cross-link precursors [9,10]. In this perspective, ZnO has a slight delay in decomposing the accelerator. The different Arrhenius plots in Figure 7c also have shown excellent linearity. From the corresponding activation energy of vulcanization in Figure 7d, the addition of MgO can largely reduce the activation energy of vulcanization. It was found that ZnO or zinc ions can stabilize the cross-link precursors through coordination bonding [11,42], whereas MgO may not stabilize the cross-link precursors and have lower stability that can more easily decompose and form cross-link precursors than ZnO-only compounds. Because of the lowering activation energies of cross-link precursors and cross-link formation, ZnO- and MgO-based binary compounds show a much higher rate of vulcanization compared to single cure activator-containing compounds (Figure 5c).

**Figure 8:**
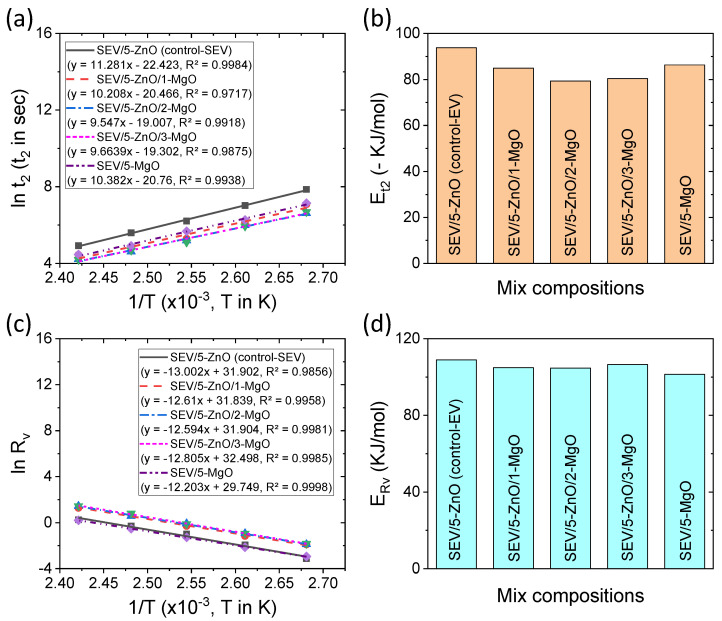
Arrhenius plots for activation energies of SEV curing systems: (**a**) Arrhenius plot with respect to scorch safety time, (**b**) activation energy for cross-link precursor formation, (**c**) Arrhenius plot with respect to true cross-linking rate, and (**d**) activation energy of cross-linking formation.

Different Arrhenius plots and the corresponding activation energies for SEV curing systems are provided in Figure 8a–d. From Figure 8a, the Arrhenius plots with respect to t_2_ are also very linear and can be useful to determine t_2_ at any arbitrary temperatures beyond the experimental temperatures. The corresponding activation energy of cross-link precursors can be found in Figure 8b. From this figure, it can be seen that like EV curing systems, the addition of MgO to the SEV curing systems can reduce the energy of activation. The lower activation energies in the binary curing systems could be due to the synergistic effect of simultaneous decomposition of accelerator by MgO and stabilization of cross-link precursors through Zn-complexation [11,42]. The Arrhenius plots with respect to R_v_ for SEV curing systems have been provided in Figure 8c. The plots are very linear and follow the Arrhenius relationship. The corresponding activation energies are provided in Figure 8d. It has been found that there are no significant differences in the activation energies after the addition of MgO in the binary cure activator systems from the control-SEV system. If we compare the activation energies in Figure 7b,d and Figure 8b,d, it can be found that a decreasing accelerator-to-sulfur ratio can reduce the activation energies of different vulcanization steps. It is believed that with increasing sulfur content in the accelerator-to-sulfur ratio, the chain length of sulfur bridges in the cross-link precursors increases and becomes easier to decompose with temperature.

**Figure 9:**
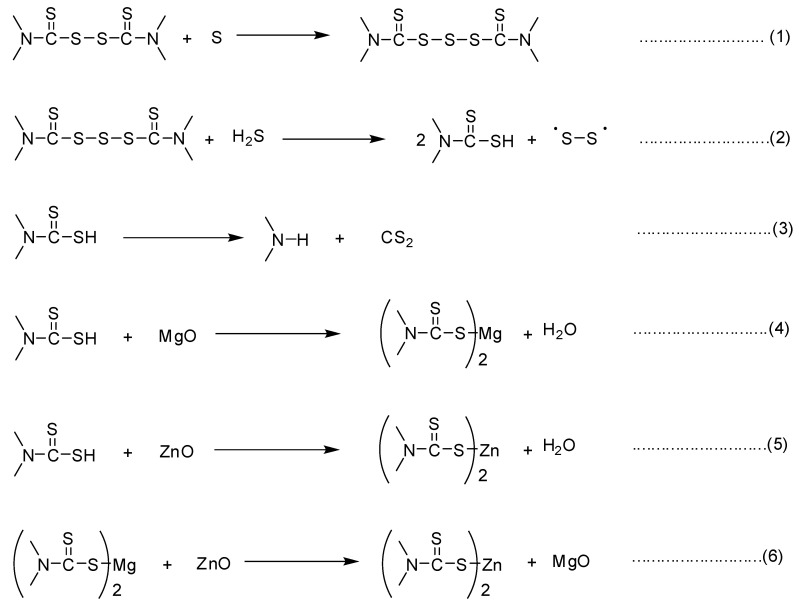
Possible interactions between accelerator, activators, and sulfur in ZnO- and MgO-based binary cure activator systems.

Joseph et al. [43] extensively discussed the detailed mechanism of accelerated sulfur vulcanization, focusing on the formation of zinc-complex compounds by accelerators and activators. Typically, stearic acid and amines can serve as ligands to these zinc complexes. While the vulcanization mechanism is well understood, it involves complex pathways that can be challenging to represent concisely. However, Alam et al. [44] simplified the mechanism of accelerated sulfur vulcanization to facilitate a better understanding of the interactions between accelerators and activators. The accelerated sulfur vulcanization in the presence of ZnO- and MgO-based binary cure activators can be understood from the different paths presented in Figure 9 (paths 1–6). From path 1, the accelerator undergoes sulfur incorporation into its molecule to form a polysulfide form [44]. This compound reacts with H_2_S from primary cross-links as shown by S-S radicals to the rubber chains, and the thiuram compound converts to its reduced state as thiocarbamic acid [44,45] through path 2. Some of the thiocarbamic acid decomposes to form free amine and CS_2_ and reduces the amount of accelerator concentration from the vulcanization system followed by path 3. However, in the presence of cure activators such as MgO or ZnO, the thiocarbamic acid is trapped as a thiocarbamate compound followed by paths 4 and 5. These thiocarbamate compounds can undergo oxidation with added sulfur and again produce sulfur cross-links as shown in paths 1 and 2. According to previous reports, it was postulated that MgO can react faster with in situ generated thiocarbamic acid compared to ZnO and results in a higher rate of vulcanization [10]. However, MgO-activated compounds produced a lower cross-link density represented by lower ΔM value. In the presence of ZnO, the rate of vulcanization is low because it slowly converts thiocarbamic acid into a zinc-based active sulfurating complex. However, it can give a higher cross-link density to the rubber compound as evidenced by higher ΔM values, which might be due to the catalytic activity of zinc-based cross-linking precursors [42]. In the presence of both MgO and ZnO, the kinetically controlled magnesium-thiocarbamate may undergo mutual interactions with ZnO to produce a thermodynamically more stable zinc thiocarbamate-type active sulfurating complex as shown by path 6.

It has been found that with increasing sulfur content from EV to SEV curing systems, the ΔM values for MgO- and ZnO-containing binary cure activator systems have lower values compared to the control-SEV system at higher temperatures. It is believed that the concentration of thermodynamically controlled zinc dithiocarbamate can be increased with temperature to enhance the ΔM value in the control-SEV vulcanizate. Moreover, it was believed that zinc ions can better stabilize the cross-link precursors than magnesium ions. During the cross-linking process, some zinc-based precursors undergo cross-linking and some regenerate new cross-link precursors. Due to such type of catalytic activity, ZnO-based cure activator always produces a higher cross-link density. On the other hand, MgO may not stabilize the cross-link precursor, but it can faster decompose the accelerator or cross-link precursor to generate polysulfide cross-links as evidenced by lower ΔM values. In the presence of both cure activators, some catalytic and non-catalytic cross-linking precursors may form and show faster decomposition of cross-link precursors with an average value of cross-link density. Moreover, it was observed that a higher amount of stable disulfide cross-links was formed in Zn/Mg-hybrid oxide rather than ZnO-only compounds with a higher number of mono-sulfidic cross-links [17]. Since disulfide links contain double the sulfur atoms than mono-sulfidic cross-links, disulfide-rich vulcanizate is obviously expected to have lower cross-link density. Although ZnO- and MgO-containing binary cure activators introduce lower ΔM values in some cases, due to the presence of disulfide cross-links in the vulcanizate, it might have better fatigue resistance properties and could be important in the tire industry to produce low-fatigue tires [46]. Thus, in the presence of MgO and ZnO, the compounds not only include better rheometric mechanical properties but also provide faster curing kinetics.

## 4. Conclusions

This article represents the reduction in vulcanization temperature by the addition of MgO as a co-cure activator in different ZnO-based curing systems such as EV and SEV. Curing study results indicate that MgO as a co-cure activator can enhance the curing kinetics at different stages of rubber vulcanization. The improvements in the curing kinetics via the addition of MgO can result in a reduced optimum curing time with improved rate of vulcanization in both curing systems. With increasing sulfur content and reducing accelerator concentrations from EV to SEV curing systems, the rate of vulcanization can be further improved, but it results in lower delta torque in the ZnO- and MgO-based co-cure activators containing vulcanizates compared to the control-SEV curing system. Hence, to obtain better mechanical properties in the ZnO- and MgO-based co-cure activating vulcanization system, the accelerator-to-sulfur ratio should not be lower than in the SEV vulcanization system. Overall, a very small amount, approximately 1 to 2 phr of MgO, can substantially reduce the vulcanization temperature by about 20 °C in comparison to the controlled vulcanizates while retaining similar vulcanization kinetics with better rheometric mechanical properties. The reduced optimum curing time by using ZnO and MgO-based binary cure activators suggests that the vulcanization of rubber can be performed even below 100 °C with a reasonable vulcanization time and better curing kinetics. The substantial improvements in the curing kinetics via the addition of MgO- to the ZnO-based curing systems might be due to reduced activation energies in different vulcanization stages followed by mutual interactions between the two cure activators.

## Figures and Tables

**Table 1:** Mixing composition of different ingredients in phr (per hundred gram of rubber).

Mix Compositions	Formulation						Vulcanization Systems (Accelerator/Sulfur)
	NR	ZnO	MgO	Stearic Acid	TMTD	Sulfur	
EV/5-ZnO (control-EV)	100	5	-	2	2.16	0.5	EV (4.32)
EV/5-ZnO/1-MgO	100	5	1	2	2.16	0.5	
EV/5-ZnO/2-MgO	100	5	2	2	2.16	0.5	
EV/5-ZnO/3-MgO	100	5	3	2	2.16	0.5	
EV/5-MgO	100	-	5	2	2.16	0.5	
SEV/5-ZnO (control-SEV)	100	5	-	2	1.44	1.5	SEV (0.96)
SEV/5-ZnO/1-MgO	100	5	1	2	1.44	1.5	
SEV/5-ZnO/2-MgO	100	5	2	2	1.44	1.5	
SEV/5-ZnO/3-MgO	100	5	3	2	1.44	1.5	
SEV/5-MgO	100	-	5	2	1.44	1.5	

## Data Availability

Data are contained within the article.

## Supplementary Materials

The following supporting information can be downloaded at: https://www.mdpi.com/article/10.3390/polym16070876/s1, Table S1: Specific rheometric mechanical and kinetic parameters at 100 °C; Table S2: Specific rheometric mechanical and kinetic parameters at 110 °C; Table S3: Specific rheometric mechanical and kinetic parameters at 120 °C; Table S4: Specific rheometric mechanical and kinetic parameters at 130 °C; and Table S5: Specific rheometric mechanical and kinetic parameters at 140 °C.
