# Supplementary material for: Enhancing Rubber Vulcanization Cure Kinetics: Lowering Vulcanization Temperature by Addition of MgO as Co-Cure Activator in ZnO-Based Cure Activator Systems
Source: Polymers (Basel). 2024 Mar 22;16(7):876. doi: 10.3390/polym16070876 (PMC11013655; doi:10.3390/polym16070876)
Supplement: Supplementary file 1 [file polymers-16-00876-s001.zip › polymers-2883531-supplementary.pdf]

Supporting Information

Enhancing rubber vulcanization cure kinetics: Lowering vulcanization temperature by addition of MgO as co-cure activator in ZnO-based cure activator systems

Md Najib Alam [1], Vineet Kumar [1], Seok U Jeong [2] Sang-Shin Park [1,*]

[1] School of Mechanical Engineering, Yeungnam University, Gyeongsan, Gyeongbuk 38541, Republic of Korea
[2] Graduate School of Mechanical Engineering, Yeungnam University, Gyeongsan, Gyeongbuk 38541, Republic of Korea
*Correspondence: S.-S. Park (pss@ynu.ac.kr)

Table S1. Specific rheometric mechanical and kinetic parameters at 100 °C.

Mix compositions	M_L (Nm)	M_H (Nm)	ΔM (Nm)	M_{300}	R_{300} (%)	t_2 (min)	t_{90} (min)
SEV/5-ZnO (control-SEV)	0.35	4.75	4.4	-	-	43.12	131.63
SEV/5-ZnO/1-MgO	0.67	5.46	4.79	5.43	0.5	19.68	48.1
SEV/5-ZnO/2-MgO	0.72	5.45	4.73	5.43	0.4	12.33	39.96
SEV/5-ZnO/3-MgO	0.82	5.58	4.76	5.55	0.5	13.38	39.44
SEV/5-MgO	0.83	3.08	2.25	3.07	0.3	21.17	59.02

Table S2. Specific rheometric mechanical and kinetic parameters at 110 °C.

Mix compositions	M_L (Nm)	M_H (Nm)	ΔM (Nm)	M_{300}	R_{300} (%)	t_2 (min)	t_{90} (min)
EV/5-ZnO (control-EV)	0.22	4.52	4.31	-	-	21.64	132.5
EV/5-ZnO/1-MgO	0.64	5.31	4.66	5.30	0.2	10.31	41.02
EV/5-ZnO/2-MgO	0.71	5.43	4.72	5.42	0.2	9.66	37.65
EV/5-ZnO/3-MgO	0.74	5.36	4.61	5.35	0.2	8.75	38.61
EV/5-MgO	0.79	3.12	2.33	3.11	0.3	14.05	37.76
SEV/5-ZnO (control-SEV)	0.31	5.38	5.07	-	-	18.62	49.93
SEV/5-ZnO/1-MgO	0.62	5.55	4.93	5.52	0.5	7.58	21.52
SEV/5-ZnO/2-MgO	0.68	5.49	4.81	5.47	0.4	6.64	17.48
SEV/5-ZnO/3-MgO	0.78	5.61	4.82	5.59	0.4	6.32	17.27
SEV/5-MgO	0.83	3.05	2.21	3.03	0.7	8.71	25.28

Table S3. Specific rheometric mechanical and kinetic parameters at 120 °C.

Mix compositions	M_L (Nm)	M_H (Nm)	ΔM (Nm)	M_{300}	R_{300} (%)	t_2 (min)	t_{90} (min)
EV/5-ZnO (control-EV)	0.19	4.78	4.58	-	-	9.13	70.48
EV/5-ZnO/1-MgO	0.55	5.39	4.84	5.37	0.4	4.5	14.35
EV/5-ZnO/2-MgO	0.61	5.51	4.9	5.50	0.2	3.99	16.11

EV/5-ZnO/3-MgO	0.67	5.52	4.86	5.49	0.5	3.71	15.65
EV/5-MgO	0.71	3.2	2.5	3.1	0.3	6.6	17.74
SEV/5-ZnO (control-SEV)	0.27	5.71	5.45	5.7	0.2	8.24	21.32
SEV/5-ZnO/1-MgO	0.55	5.44	4.89	5.39	0.9	3.29	9.12
SEV/5-ZnO/2-MgO	0.62	5.47	4.84	5.38	1.6	2.97	7.62
SEV/5-ZnO/3-MgO	0.72	5.65	4.93	5.56	1.6	2.74	7.61
SEV/5-MgO	0.82	3.24	2.43	3.17	2.1	4.86	12.6

Table S4. Specific rheometric mechanical and kinetic parameters at 130 °C.

Mix compositions	M_L (Nm)	M_H (Nm)	ΔM (Nm)	M_{300}	R_{300} (%)	t_2 (min)	t_{90} (min)
EV/5-ZnO (control-EV)	0.17	4.34	4.18	-	-	4.48	16.56
EV/5-ZnO/1-MgO	0.46	5.09	4.62	5.05	0.7	2.37	7.09
EV/5-ZnO/2-MgO	0.52	5.25	4.73	5.22	0.6	2.02	6.2
EV/5-ZnO/3-MgO	0.58	5.28	4.7	5.26	0.4	2.01	7.55
EV/5-MgO	0.64	3.37	2.72	3.12	7.4	3.26	8.67
SEV/5-ZnO (control-SEV)	0.23	5.84	5.6	5.81	0.5	4.48	11.13
SEV/5-ZnO/1-MgO	0.52	5.41	4.88	5.23	3.3	2	4.21
SEV/5-ZnO/2-MgO	0.57	5.38	4.81	5.22	3.0	1.66	4.05
SEV/5-ZnO/3-MgO	0.65	5.58	4.92	5.39	3.4	1.8	3.73
SEV/5-MgO	0.75	3.23	2.48	3.08	4.6	2.26	5.95

Table S5. Specific rheometric mechanical and kinetic parameters at 140 °C.

Mix compositions	M_L (Nm)	M_H (Nm)	ΔM (Nm)	M_{300}	R_{300} (%)	t_2 (min)	t_{90} (min)
EV/5-ZnO (control-EV)	0.17	4.36	4.18	4.35	0.2	2.36	8.54
EV/5-ZnO/1-MgO	0.39	5.04	4.64	4.97	1.4	1.73	4.24
EV/5-ZnO/2-MgO	0.43	5.11	4.68	5.06	1.0	1.57	3.71
EV/5-ZnO/3-MgO	0.48	5.12	4.64	5.06	1.2	1.41	3.57
EV/5-MgO	0.57	3.27	2.71	2.75	17.1	1.85	4.52
SEV/5-ZnO (control-SEV)	0.22	5.5	5.28	5.48	0.4	2.27	5.89
SEV/5-ZnO/1-MgO	0.42	5.23	4.81	4.89	6.5	1.42	2.61
SEV/5-ZnO/2-MgO	0.51	5.24	4.73	4.84	7.6	1.12	2.11
SEV/5-ZnO/3-MgO	0.61	5.49	4.88	5.06	7.8	1.1	2.11
SEV/5-MgO	0.67	3.28	2.61	2.88	12.2	1.44	3.32
